# A Scoping Review on Cognition in Myelodysplastic Syndromes: Advances and Challenges

**DOI:** 10.3390/medsci13010015

**Published:** 2025-02-07

**Authors:** Anna Tsiakiri, Konstantinos Frigkas, Pinelopi Vlotinou, Menelaos Papoutselis, Foteini Christidi, Efstratios Karavasilis, Ioannis Kotsianidis, Nikolaos Kourkoutsakis, Konstantinos Vadikolias, Konstantinos Liapis

**Affiliations:** 1 Department of Neurology, Democritus University of Thrace, 68100 Alexandroupolis, Greece; christidi.f.a@gmail.com (F.C.); kvadikol@med.duth.gr (K.V.); 2 Department of Radiology, Democritus University of Thrace, 68100 Alexandroupolis, Greece; kfrigkas@med.duth.gr (K.F.); ncourcou@med.duth.gr (N.K.); 3 Department of Occupational Therapy, University of West Attica, 12243 Athens, Greece; pvlotinou@uniwa.gr; 4 Department of Hematology, Democritus University of Thrace, 68100 Alexandroupolis, Greece; menelaospapoutselis@yahoo.co.uk (M.P.); ikotsian@med.duth.gr (I.K.); koliapi@med.duth.gr (K.L.); 5 Medical Physics Laboratory, Democritus University of Thrace, 68100 Alexandroupolis, Greece; ekaravas@med.duth.gr

**Keywords:** myelodysplastic syndrome, cognitive impairment, chemo brain, cancer-related cognitive impairment, hematopoietic stem cell transplantation

## Abstract

Background/Objectives: Myelodysplastic syndromes (MDS) are clonal hematopoietic disorders characterized by ineffective hematopoiesis and a risk of progression to acute myeloid leukemia (AML). Cognitive impairments, including deficits in memory, attention, and executive function, are frequently reported in MDS patients. These impairments are linked to systemic inflammation, neurotoxic treatment effects, and the psychological burden of chronic disease. This review synthesizes existing evidence on cognitive dysfunction in MDS, highlighting knowledge gaps and opportunities for future research. Methods: A scoping review was conducted following PRISMA-ScR guidelines. MEDLINE and Scopus databases were searched for studies examining cognition in MDS patients, using terms like “myelodysplastic syndromes” and “cognition.” Inclusion criteria were original, English-language studies reporting cognitive outcomes in MDS. Reviews, animal studies, and abstracts were excluded. Data on study design, patient characteristics, cognitive tests, and outcomes were extracted and descriptively analyzed. Results: The review included 25 studies involving 2390 patients with hematologic malignancies, 493 of whom had MDS. Key findings identified cognitive deficits primarily in attention, executive function, and memory. Systemic inflammation and treatment-related neurotoxicity were significant contributors, with older age compounding these effects. Longitudinal studies demonstrated persistent cognitive challenges post-treatment, though the severity varied by patient demographics and therapeutic regimens. Conclusions: Cognitive impairments in MDS are multifactorial and significantly impact patients’ quality of life. Current research highlights the need for routine cognitive assessments and targeted interventions. Future studies should focus on longitudinal designs to explore specific cognitive domains and develop therapeutic strategies to mitigate cognitive decline.

## 1. Introduction

Myelodysplastic syndrome (MDS) is a collection of clonal hematopoietic stem cell disorders characterized by ineffective hematopoiesis, leading to one or more cytopenias, and a propensity for transformation to AML. The clinical presentation often includes anemia, necessitating frequent blood transfusions; thrombocytopenia, resulting in bleeding tendencies; and neutropenia, increasing the risk of infections. The disease predominantly affects older adults, with an incidence increasing significantly in individuals over 70 years old. The heterogeneity in clinical manifestations and disease progression makes MDS a challenging condition to manage [1].

The cognitive aspects of MDS are gaining attention as patients and clinicians report cognitive deficits during and after treatment. These impairments, commonly termed cancer-related cognitive impairment (CRCI), refer to a clinically recognized phenomenon characterized by difficulties in memory, attention, executive function, and processing speed. CRCI is frequently associated with chemotherapy (i.e., chemotherapy-induced cognitive impairment or CICI) and other cancer treatments. The exact mechanisms underlying these cognitive changes are not fully understood, but they are thought to involve a combination of direct neurotoxic effects of treatments, systemic inflammatory responses, and the psychological stress of managing a chronic and potentially progressive disease [2]. Structural brain changes, such as leukoaraiosis, which are indicative of chronic small vessel disease and systemic inflammation, have been shown to significantly impair cognitive function, particularly in domains such as executive function and attention [3]. Similar mechanisms may underlie cognitive deficits observed in patients with MDS, emphasizing the need for further exploration of inflammatory and vascular contributions to these impairments. Treatments for MDS include hematopoietic stem cell transplantation (HSCT), which is associated with neurocognitive side effects, such as treatment-induced neurotoxicity and cognitive dysfunction [4].

Hypomethylating agents, particularly in older patients, have been associated with cognitive changes, likely due to their impact on epigenetic regulation within the central nervous system [5]. The use of supportive therapies, such as erythropoiesis-stimulating agents (ESAs) and granulocyte colony-stimulating factors (G-CSF), also raises concerns about cognitive side effects, though these are less well-documented [6]. A number of studies have begun to elucidate the cognitive challenges faced by patients with MDS. Research has shown that cognitive impairment in these patients can be multifactorial. For example, a review by Williams et al. [2] indicated that survivors of hematological malignancies, including MDS, often experience significant cognitive deficits, particularly after intensive treatments such as HSCT. These deficits are thought to arise from multiple factors, including treatment-related neurotoxicity, psychological stress, and systemic inflammation. Other studies have investigated the biological underpinnings of these cognitive impairments. For instance, Lange et al. noted that chemotherapy could induce structural changes in the brain, such as reduced gray matter volume and altered connectivity, which correlate with observed cognitive deficits. Moreover, genetic polymorphisms, such as those in APOE-4 and COMT, have been associated with an increased risk of cognitive impairment in cancer patients, suggesting a potential genetic predisposition [7].

Clonal hematopoiesis of indeterminate potential (CHIP), an age-related premalignant condition, is increasingly recognized for its influence beyond hematologic malignancies. Characterized by somatic mutations in hematopoietic stem cells that provide a clonal advantage, CHIP has been associated with systemic inflammation, cerebrovascular risks, and cognitive outcomes. Recent findings suggest that CHIP may exert protective effects against cognitive impairments in specific populations. For example, in chronic kidney disease (CKD) patients, CHIP was linked to a reduced risk of impairments in attention and executive function, though no significant associations were observed for global cognition or verbal memory [8]. These findings align with emerging evidence suggesting that CHIP-driven biological mechanisms, such as enhanced phagocytic function in microglia, may modulate neuroinflammatory pathways and cognitive resilience. In hematologic malignancies like MDS, systemic inflammation is similarly implicated in cognitive decline. This raises intriguing questions about whether CHIP-related protective mechanisms extend to MDS patients, potentially modifying their cognitive trajectories [9,10,11].

Despite these insights, significant gaps remain in understanding the full extent and nature of cognitive impairments in MDS patients. The literature is sparse on comprehensive, longitudinal studies that track cognitive function from diagnosis through various treatment stages and into survivorship. Additionally, the interplay between different treatments, patient demographics, and cognitive outcomes is not well-defined. Many studies have focused on broad cognitive domains, such as memory, attention, and executive function, emphasizing that these domains are affected differently depending on patient characteristics, treatment type, and disease stage. While considerable progress has been made in understanding MDS and its treatment, the cognitive aspects of this disease remain underexplored. This review will contribute to the field by offering a comprehensive overview of the current state of knowledge, identifying critical gaps, and proposing directions for future research to improve patient care and quality of life.

## 2. Materials and Methods

This scoping review is part of a broader research protocol investigating cognitive functions in patients with MDS. The study is being conducted at the University Hospital of Alexandroupolis and has been approved by the Scientific Council (Approval Number: ΕΣ7/Θ3/07-04-2022). While the review itself was not pre-registered, it adheres to the objectives and methodology outlined in the approved research protocol.

### 2.1. Literature Search

This scoping review used the 22-item Preferred Reporting Items for Systematic Reviews and Meta-Analyses extension for Scoping Reviews (PRISMA-ScR) [12]. Our study’s methods were a priori designed. Two investigators (A.T. and K.F.) conducted a literature search of two databases (MEDLINE and SCOPUS) to trace all relevant studies published. Using the advanced search function, the search string included compound keywords, using the BOOLEAN expression “myelodysplastic syndrome” AND “cognition”. Papers containing any of these terms in their titles, abstracts, or keywords were considered. All retrieved articles were also manually searched for further potentially eligible articles. Any disagreement regarding the screening or the selection process was further discussed with a third investigator (F.C.) until a consensus was reached.

### 2.2. Selection Criteria

We included only original full-text articles published in the English language. Secondary analyses, reviews, guidelines, meeting summaries, comments, unpublished abstracts, clinical trials, and studies conducted with animals were excluded.

### 2.3. Data Extraction

Data extraction was carried out using a predefined form created in Excel. We documented the authors, the year of publication, the type of study, the characteristics of the patient and reference groups, the timing and measures of neuropsychological testing, and the main cognitive findings of the study.

### 2.4. Data Analysis

No statistical analysis or meta-analysis was performed due to the high heterogeneity observed among studies. However, we conducted a descriptive analysis to summarize key study characteristics and cognitive outcomes (Table 1).

## 3. Results

### 3.1. Database Search

**Figure 1:**
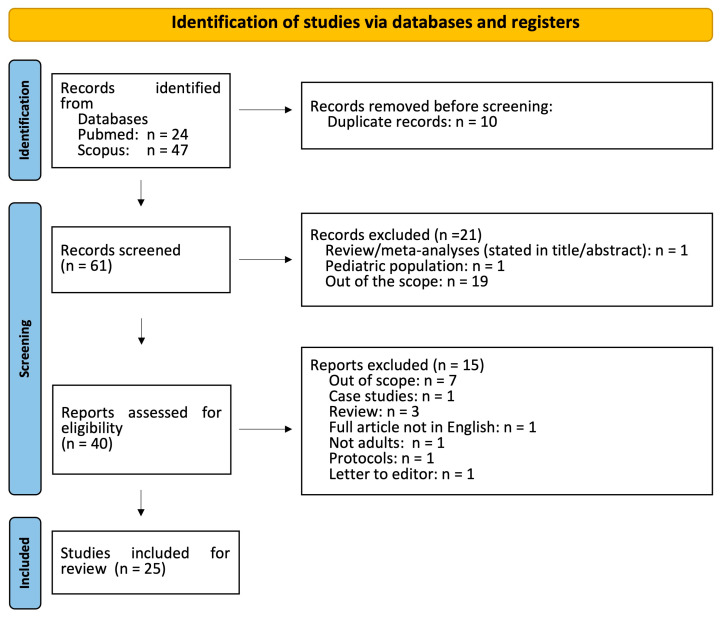
Study flow diagram (PRISMA flowchart).

Overall, 71 records (Scopus: 47; PubMed: 24) were retrieved from the database search. After removing duplicates, 61 studies were included. Based on the inclusion/exclusion criteria, all irrelevant studies were excluded; therefore, a total of 40 articles were selected. After screening the full texts of the articles, 25 studies were judged to be eligible for inclusion (Figure 1). All studies included in the review are presented in Supplementary Table S1.

### 3.2. Study Origin

The dataset provides a comprehensive global view of research into the neuropsychological effects associated with hematological diseases, showcasing contributions from multiple regions. The United States is prominently represented with thirteen studies [13,14,15,16,17,18,19,20,21,22,23,24,25], Europe contributes seven studies [26,27,28,29,30,31,32]. Contributions from other regions include Brazil [33], Asia [34], Israel [35], Egypt [36], and Australia [37]. These varied research efforts highlight the diverse methodologies and focal points of studies conducted around the world, enhancing our understanding of the cognitive impacts of severe hematological conditions across different patient demographics and healthcare settings.

### 3.3. Study Design

In the reviewed literature, eight studies utilized a cross-sectional design, providing a single-time-point analysis, while seventeen studies employed a longitudinal design, allowing for the observation of changes over time through multiple data collection points.

### 3.4. Patient Groups and Demographic Profiles

The research encompassed a comprehensive analysis of 2390 patients diagnosed with various hematologic malignancies. This cohort consisted of 1226 male patients and 881 female patients, indicating a slight male dominance. Notably, a significant subset of this population, totaling 493 individuals, was identified with MDS. The analysis of the patient data revealed that the average age is 67.01 years, with a median age of 72 years. The age distribution among the patients shows a standard deviation of 13.79 years, indicating a moderate spread around the average. The youngest patient is recorded at 39 years old, while the oldest is 77.2 years. These statistics provide a snapshot of the age demographics within the study, highlighting a predominantly older population, which is critical for understanding the patient characteristics and potential implications for healthcare outcomes.

### 3.5. Reference Groups

According to the data, two studies utilized a control group. In the first study [17], the control group consisted of 75 individuals, including 42 females and 33 males, with an average age of 52.97 years and a standard deviation of 14.91 years. The second study [31] included 465 individuals in the control group, with a mixed gender composition and an average age of 58.9 years (standard deviation of 8.6 years).

### 3.6. Materials Employed for Neuropsychological Assessment

**Figure 2:**
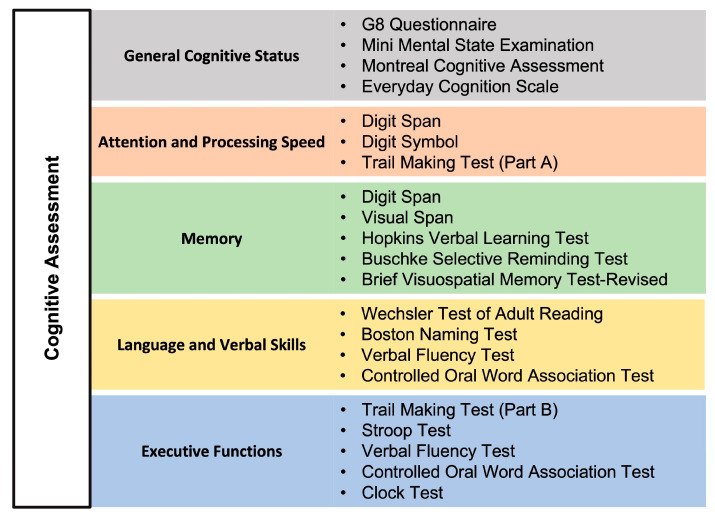
Cognitive domains most commonly assessed in the studies of the current review and related neuropsychological tests.

**Figure 3:**
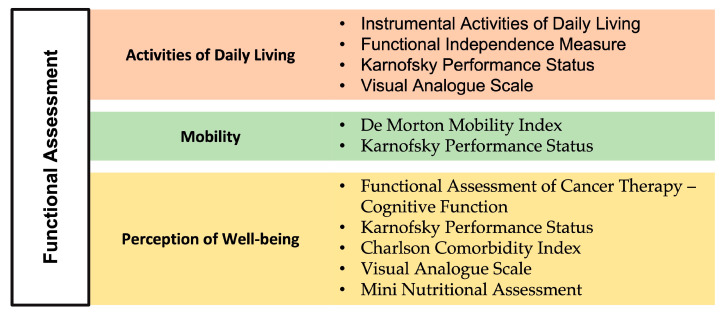
Functional domains most commonly assessed in the studies of the current review and related scales and questionnaires.

**Figure 4:**
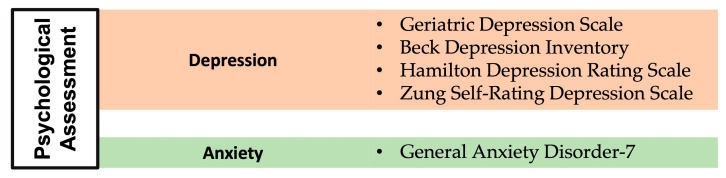
Emotional domains most commonly assessed in the studies of the current review and related questionnaires.

In this section, we provide an overview of the most commonly used neuropsychological tools across the studies included in this review, emphasizing their utility in evaluating cognitive, functional, and psychological domains in patients with MDS. The assessments are categorized into three primary domains: cognitive evaluations, functional assessments, and psychological and mood assessments. Cognitive assessments were utilized to evaluate general cognitive status, memory, attention, processing speed, language and verbal skills, and executive functions. Figure 2 categorizes these tools by the cognitive domains they evaluate, providing a structured view of their application in MDS research. Functional assessments primarily focused on evaluating activities of daily living, mobility, and perception of well-being. Figure 3 illustrates the organization of functional assessments into these core domains, highlighting their relevance to understanding the broader impact of MDS on patient quality of life. Psychological and mood assessments were used to measure depression, anxiety, and other emotional factors that may influence cognitive function and recovery. Figure 4 categorizes these tools based on the psychological and mood factors they target.

### 3.7. Neuropsychological Performance on Specific Cognitive Domains

**Figure 5:**
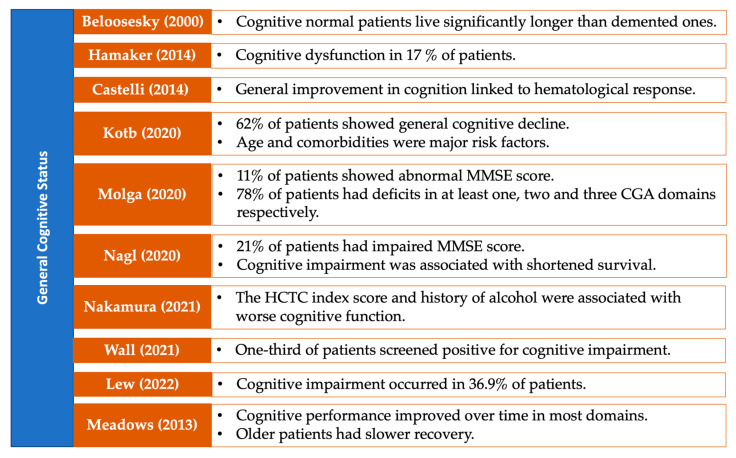
Representative studies [15,19,22,24,26,28,29,35,36,37] reporting general cognitive status in patients with MDS and other hematological malignancies.

**Figure 6:**
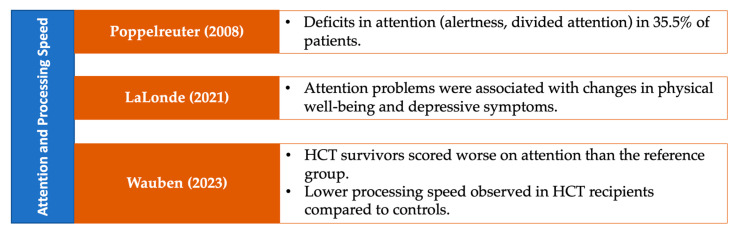
Representative studies [23,30,31] reporting attention and processing speed in patients with MDS and other hematological malignancies.

**Figure 7:**
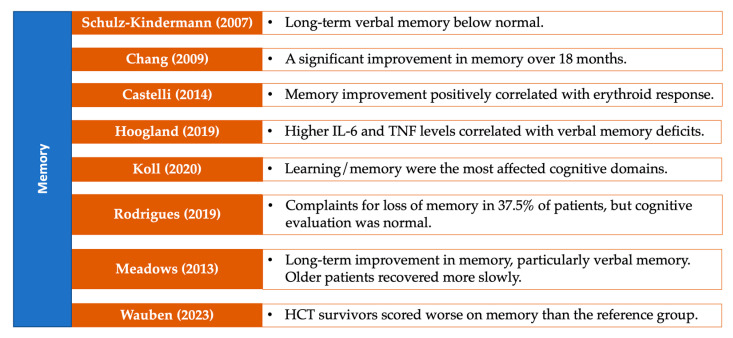
Representative studies [13,16,24,25,28,31,32,33] reporting memory in patients with MDS and other hematological malignancies.

**Figure 8:**
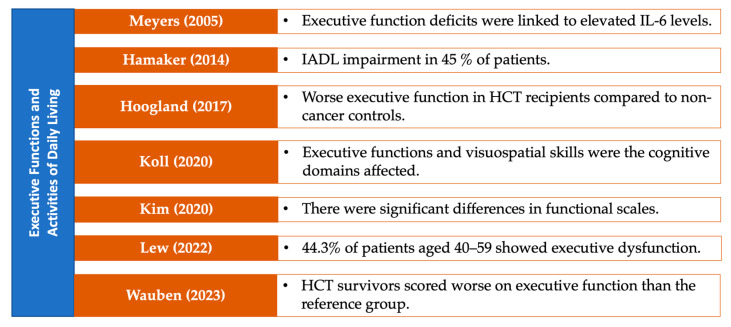
Representative studies [13,14,15,16,26,31,34] reporting executive functions and activities of daily living in patients with MDS and other hematological malignancies.

The results in this section are visually summarized in a series of figures that categorize the studies based on the cognitive domains assessed in patients with MDS. Each figure highlights key findings from the reviewed studies, focusing on specific cognitive domains, including general cognitive status (Figure 5), attention and processing speed (Figure 6), memory (Figure 7), and executive functions (Figure 8). Together, these figures (Figure 5, Figure 6, Figure 7 and Figure 8) provide a comprehensive, domain-specific view of the cognitive challenges faced by patients with MDS, facilitating a clearer understanding of the heterogeneity of findings across the included studies.

### 3.8. Time of Neuropsychological Testing

Baseline and Follow-up Testing: This cluster includes studies where neuropsychological (NPS) testing was conducted both at baseline and at various follow-up points. Scherwath [27] performed assessments before conditioning (T0), 100 days post-transplant (T1), and 12 months later (T2). Similarly, Koll [16] evaluated patients before and after HCT. Hoogland [17] tested participants before transplantation and then again at three months and one year post-transplantation. Meadows [24] tracked cognitive outcomes from baseline through 12 and 18 months post-treatment. Schulz-Kindermann [32] conducted evaluations two weeks before admission (baseline, T0) and 100 days post-transplant (T1).

Pre- and Post-Intervention: Rodrigues [33] uniquely focused on assessments conducted in hospital at least one week before admission for allo-HSCT. This approach highlights the evaluation of patients in the context of hospital-based interventions and their immediate pre-intervention states.

Post-Treatment Only: Several studies concentrated on post-treatment evaluations without a prior baseline measurement. Hoogland [13] and Castelli [28] examined patients before and after HCT and at various points post-treatment, such as 90 days or eight weeks after the treatment, respectively. Nagl [29] assessed patients within 14 days after initial diagnosis, while Loh [18] included both pre- and post-intervention testing. Nakamura [19] focused on assessments after admission and before transplantation. LaLonde [23] covered the period pre-HSCT and at 30 and 100 days post-HSCT. Chang [25] provided evaluations at enrollment and followed up at 12 and 18 months post-HSCT. Wauben [31] observed long-term survivors of stem cell transplantation, monitoring for cognitive changes after two years. Molga [37] assessed patients after the treatment decision was made. These studies emphasize the cognitive outcomes following medical interventions and during recovery phases.

During Hospitalization: Hamaker [26] stands alone in this cluster, focusing on patients who were newly diagnosed and assessed during hospitalization. This study captures the immediate cognitive status of patients upon receiving their diagnosis and starting treatment.

Long-term Follow-up: The long-term follow-up cluster includes studies that extended their observation period beyond immediate post-treatment phases. Poppelreuter [30] conducted NPS testing at the start and end of inpatient rehabilitation, with a follow-up six months later, providing a comprehensive view of recovery. Beloosesky [35] monitored patients over a wide range of 1 to 70 months, highlighting prolonged outcomes. Kotb [36] followed patients who completed chemotherapy from six months to two years prior, focusing on long-term cognitive effects. This cluster provides valuable data on the enduring impact of medical treatments on cognitive functions.

Special Timing: This diverse cluster includes studies with unique timing of assessments. Kim [34] conducted testing at enrollment and discharge, capturing changes over a specific treatment period. Meyers [14] focused on pretreatment evaluations and then one month later, providing early insights into treatment effects. Lew [15] examined patients at new patient evaluation and again one week before HCT, highlighting preparatory cognitive status. Chang [21] assessed patients initially during treatment initiation and then after 12 months, providing a long-term perspective. Wall [22] included patients referred to the CARE clinic, focusing on specialized care contexts. This cluster reflects diverse study designs that address specific clinical questions and patient populations.

## 4. Discussion

### 4.1. Cognitive Decline in MDS and Other Hematological Malignancies: General Findings and the Role of Demographic Factors and Other Patient-Related Characteristics

Recent studies have increasingly focused on the prediction of cognitive decline among patients with MDS and other hematological malignancies, recognizing the need for early identification and intervention. Hamaker et al. [26] explored the utility of geriatric assessments, particularly the G8 questionnaire, in predicting cognitive decline. While the G8 lacked strong discriminative power, it highlighted a high prevalence of geriatric conditions in elderly patients, suggesting a potential link to cognitive decline. Rodrigues et al. [33] expanded on this by utilizing Comprehensive Geriatric Assessment (CGA) tools to identify syndromes associated with dementia in MDS patients. The study found that CGA was effective in detecting frailty and other cognitive risk factors, providing a valuable framework for predicting dementia onset.

In the context of hematopoietic stem cell transplantation (HSCT), Kim et al. [34] found that most patients did not experience significant cognitive decline post-transplantation, indicating a relatively low immediate risk for dementia. However, Hoogland et al. [13] noted that increases in inflammatory markers, such as IL-6 and sTNF-RII, were correlated with cognitive deterioration post-hematopoietic cell transplantation (HCT), suggesting inflammation as a potential mechanism linking hematological treatments to cognitive decline. Meyers et al. [14] further emphasized the high prevalence of cognitive impairments in patients with AML and MDS following intensive treatments, underlining the necessity of early neuropsychological evaluation.

Cognitive decline in patients with MDS and other hematologic malignancies is influenced by various factors, including age, specific diagnoses, and biological markers. A study revealed that elderly patients with these malignancies often have a high prevalence of geriatric conditions, which are closely linked to cognitive impairments [26]. This suggests that advancing age may exacerbate cognitive challenges in this patient population. Another study demonstrated that age is a significant determinant of cognitive decline, with older patients exhibiting higher rates of impairment [15]. Of note, systemic inflammation, which is directly linked to MDS, exacerbates cognitive deficits independently of age. Despite evidence of age-related impairments in MDS, another study found that many patients retained a significant degree of independence in their daily activities. This indicates that while cognitive dysfunctions are present, they do not always severely impact functional autonomy. This resilience in daily functioning, despite cognitive difficulties, highlights the variability in how cognitive impairments manifest among different patients [33].

Further, the research conducted by Kim [34] noted no significant differences in cognitive assessments, specifically using the Visual Analogue Scale (VAS), in patients undergoing hematopoietic stem cell transplantation (HSCT). This finding suggests that the cognitive impact may not differ significantly across MDS and other types of hematologic malignancies in these clinical settings, indicating a common cognitive trajectory despite differing underlying diseases. A significant biological factor contributing to cognitive changes is inflammation. Increased levels of inflammatory markers such as IL-6 and sTNF-RII from pre- to post-HCT were associated with cognitive alterations [13]. This points to a potential mechanistic link between systemic inflammation and cognitive decline, suggesting that inflammation might be a therapeutic target for mitigating cognitive issues in these patients. The mechanisms underlying cognitive impairments in MDS patients are thought to involve systemic inflammation and treatment-related neurotoxicity, among others. Elevated levels of inflammatory markers, such as IL-6, contribute to blood-brain barrier dysfunction, allowing neurotoxic agents to penetrate the central nervous system and exacerbate neuronal damage. Chronic inflammation promotes oxidative stress, creating a feedback loop that accelerates neuronal injury and cognitive decline. Chemotherapeutic agents, particularly hypomethylating agents, exacerbate these effects by inducing systemic inflammation and disrupting epigenetic regulation within the central nervous system. Lastly, the study by Meyers et al. [14] specifically highlighted that patients with AML or MDS are particularly prone to experiencing cognitive impairments. This observation underscores a distinct cognitive profile associated with these diagnoses, characterized by a high symptom burden and significant cognitive deficits.

These studies collectively highlight the importance of comprehensive and routine cognitive assessments in patients with hematological malignancies. Early detection of cognitive decline allows for timely interventions, potentially mitigating the progression to dementia. The findings call for further research into standardized assessment protocols and the exploration of biological markers as predictive tools. The interplay between MDS-related factors, age, comorbidities, and treatment regimens complicates the assessment of cognitive impairments. Therefore, the overlapping factors influencing cognition, such as age, treatment and inflammatory status, highlight the importance of future research designed to disentangle the individual and combined contributions of these variables to cognitive decline in MDS patients. While some patients maintain functional independence, others experience notable cognitive challenges, particularly in cases like AML and MDS, where the cognitive profile is more pronounced.

### 4.2. Specific Cognitive Impairments in MDS and Other Hematological Malignancies

The studies reviewed reveal that MDS and other hematologic malignancies impact specific cognitive abilities differently, depending on the type of malignancy and treatment. Notably, patients with MDS exhibit a high prevalence of geriatric conditions with significant impairments in attention and executive function. This suggests that these cognitive domains are particularly vulnerable in elderly patients with MDS, likely exacerbated by age-related cognitive decline [26]. Rodrigues [33] highlighted that while patients with MDS maintained a level of independence in daily activities, they still experienced cognitive impairments such as reduced processing speed. This decline suggests challenges in the rapid processing of information, impacting their ability to respond swiftly in various situations. Similar findings were presented in other clinical contexts, such as stroke populations [38]

Following HSCT, cognitive decline—especially in memory and executive function—is common in MDS patients. Systemic inflammation is considered a key factor in these deficits [13,27]. Other studies [14,16] reported the same findings among patients with AML and MDS. These impairments were observed consistently across different stages of treatment, indicating persistent cognitive challenges that affect communication skills and memory retention. Furthermore, researchers [15,28] identified cognitive impairments in a notable proportion of patients, with Lew et al. [15] noting a 23% prevalence rate, while Castelli et al. [28] highlighted variations in specific cognitive test performances, indicating a diverse range of cognitive deficits across different hematologic malignancies.

Findings of research discussed the impact of comorbidities, particularly dementia, on cognitive function. Nagl et al. [29] found that 3.3% of patients had dementia, especially those with prior cerebrovascular issues, while Beloosesky et al. [35] noted no significant differences in certain cognitive measures between demented and non-demented patients, suggesting other contributing factors to cognitive outcomes. LaLonde et al. [23] observed that patient-reported cognitive complaints often did not align with objective test results, highlighting a discrepancy between perceived and actual cognitive performance. Similarly, Wauben et al. [31] found a 34% prevalence of cognitive dysfunction, illustrating the varied experiences of cognitive impairment among patients.

Long-term cognitive effects were noted by Meadows et al. [24] and Schulz-Kindermann et al. [32], particularly in verbal long-term memory, which was frequently below the norm. This indicates that cognitive impairments can persist long after initial treatment, affecting patients’ quality of life. In contrast, no significant changes in pain perception or related cognitive functions in HSCT patients were found [34], while cognitive impairments post-allogeneic hematopoietic cell transplantation are influenced by the specifics of the treatment regimen [13]. Treatments for MDS, including HSCT and hypomethylating agents, have been associated with both short- and long-term cognitive impairments, with significant variability in severity among patients. Long-term consequences of these treatments include persistent deficits in memory, attention, and executive function, which can extend for years after treatment completion. Studies have shown that HSCT recipients may experience prolonged impairments in processing speed and attention, even two years post-transplant, while hypomethylating agents have been linked to sustained disruptions in epigenetic regulation, potentially contributing to long-term neurocognitive changes. Finally, specific cognitive domains such as semantic fluency and complex information processing were identified as particularly affected and significant impairments in these areas, with a substantial portion of patients displaying cognitive dysfunction, were detected [21,22].

### 4.3. Cognitive Profile in MDS and Other Hematological Malignancies: Are These Cognitive Deficits Reversible over Time?

In the context of hematologic malignancies, the potential for reversing or improving cognitive impairments is influenced by several critical factors, including the underlying causes of the impairments, the specific type of malignancy, the modalities of treatment administered, and the overall health status of the patient. Cognitive impairments in these patients are often associated with treatable conditions such as depression, anxiety, and sleep disorders, which are prevalent among cancer patients. Addressing these underlying conditions through medications, therapy, or lifestyle interventions can lead to improvements in cognitive functions. For instance, managing mental health issues can alleviate symptoms of cognitive decline, thereby enhancing patients’ quality of life.

Inflammation has been identified as a significant contributor to cognitive decline. Research, including studies by Hoogland et al. [13] and Scherwath et al. [27], underscores the role of inflammatory responses in exacerbating cognitive impairments, especially in memory and executive functions. Anti-inflammatory treatments or strategies to manage these inflammatory responses may mitigate such cognitive declines. Cognitive rehabilitation and training programs have shown promise in improving specific cognitive functions. These programs typically involve exercises designed to enhance memory, attention, and executive functions. Studies [33] indicate that even when patients experience cognitive slowdowns, targeted interventions can help maintain some level of functional independence. Such interventions are crucial in helping patients manage everyday tasks and maintain a better quality of life.

Pharmacological interventions also play a role in addressing cognitive impairments. Cognitive enhancers, such as donepezil, and neuroprotective agents may provide benefits, particularly for patients experiencing significant cognitive deficits. While the effectiveness of these treatments varies, research [15,36] suggests that pharmacological approaches can be a valuable component of a comprehensive treatment plan for cognitive impairments. Overall physical and mental health improvements are essential for better cognitive outcomes. Regular physical activity and a balanced diet are associated with improved cognitive functions [23,31]. Additionally, maintaining a supportive mental health environment can significantly aid in cognitive recovery, underscoring the need for holistic care approaches.

The management of treatment-related side effects, such as those from chemotherapy and radiotherapy, is another crucial aspect. These treatments can lead to cognitive impairments often referred to as “chemo brain” or “chemo fog”. According to studies [14,24], addressing these side effects can facilitate recovery of cognitive functions, highlighting the importance of supportive care during and after cancer treatments. Long-term cognitive effects, particularly in areas such as verbal memory and executive function, require ongoing monitoring and intervention. Studies [32,35] emphasize the need for continuous cognitive assessments to address these long-term impacts effectively. This approach ensures that any persistent or emerging cognitive issues are promptly identified and managed. Furthermore, research [21,22] has shown significant impairments in specific cognitive domains, such as semantic fluency and complex information processing, especially in patients undergoing aggressive treatments. These findings highlight the necessity for targeted interventions tailored to address specific cognitive deficits, which can vary widely among patients. Lastly, the discrepancy between perceived and actual cognitive performance underscores the importance of objective cognitive assessments. Patients’ subjective reports of cognitive impairments may not always align with clinical findings, making it essential to rely on standardized cognitive testing to accurately determine the extent of cognitive deficits and the effectiveness of interventions [23].

### 4.4. Advances in Understanding Cognitive Impairments

A notable advancement in the field is the comprehensive use of geriatric and neuropsychological assessments to identify cognitive deficits. CGA can effectively identify cognitive risks by uncovering geriatric syndromes, such as frailty and functional decline. These tools allow for a nuanced understanding of the cognitive landscape in patients with MDS, providing early indications of potential dementia [15,33]. Another significant advancement is the identification of treatment-related cognitive effects. Studies [14,36] detailed the cognitive decline associated with chemotherapy in patients with AML and MDS. These studies reported impairments in areas such as memory and executive function, highlighting the neurotoxic impact of intensive treatment regimens. The recognition of chemotherapy’s cognitive side effects has prompted further investigation into protective strategies and the modification of treatment protocols to mitigate these effects. Furthermore, research has advanced the understanding of the biological underpinnings of cognitive decline. This study linked elevated inflammatory markers, specifically IL-6 and sTNF-RII, with cognitive impairments post-hematopoietic cell transplantation (HCT). This discovery points to systemic inflammation as a potential driver of cognitive decline, suggesting that anti-inflammatory treatments could play a role in preserving cognitive function [13].

While the clinical aspects of cognitive impairment in MDS have been a primary focus, social and psychological factors play an equally important role in cognitive recovery and overall quality of life. For example, limited social support, as reported by 21% of patients in Hamaker et al. [26], has been linked to poorer cognitive outcomes. Caregiver involvement and access to community resources may mitigate these effects by providing emotional and practical support. Psychological factors, such as depression and anxiety, are also critical contributors. Studies [33] highlight that depressive symptoms, present in 24% of patients, are strongly associated with impairments in memory and executive function. The bidirectional relationship between cognitive deficits and psychological distress suggests that interventions addressing mental health could improve cognitive recovery.

### 4.5. Challenges in the Field

Despite these advancements, significant challenges persist. One major challenge is the heterogeneity of cognitive outcomes among patients. This variability is influenced by a multitude of factors, including patient age, disease type, treatment modality, and baseline cognitive status. Such diversity complicates the establishment of uniform guidelines for cognitive assessment and management, as the cognitive trajectory can vary widely between patients [18,21]. The complexity of assessing cognitive impairment is further exacerbated by the lack of standardized assessment tools. Commonly used cognitive tests might not be adequately sensitive to detect subtle changes, especially in patients with complex clinical profiles [19,20]. This limitation underscores the need for more refined and sensitive assessment instruments that can accurately reflect the nuanced cognitive challenges faced by these patients. Another significant challenge is the under-recognition of cognitive impairments. Cognitive deficits are often overshadowed by more pressing physical health concerns, leading to their underdiagnosis and undertreatment [27,30]. This issue is particularly pronounced in older adults, where cognitive symptoms might be mistakenly attributed to aging rather than underlying pathology. The importance of longitudinal studies to track cognitive changes over time has also been another challenge [22,31]. Consistent monitoring is essential to gain insights into the long-term cognitive outcomes associated with hematological malignancies and their treatments, thereby informing more effective intervention strategies.

To address these challenges, a multifaceted approach is necessary. There is a pressing need for the development of standardized cognitive assessment protocols that are sensitive to the specific needs of hematological malignancy patients. This includes incorporating biomarkers, like inflammatory markers or hemoglobin levels, which may provide additional insights into cognitive status. Moreover, the healthcare community must prioritize the recognition and management of cognitive impairments. This could involve training healthcare providers to recognize cognitive symptoms and developing targeted cognitive rehabilitation programs [24,32]. Tailored interventions that consider the psychological and social aspects of cognitive decline can significantly enhance patient quality of life. In summary, while significant progress has been made in understanding cognitive impairments in hematological malignancies, addressing the identified challenges is crucial. Future research should focus on refining assessment tools, understanding the role of biological markers, and developing comprehensive, patient-centered care strategies. These efforts will be essential in improving cognitive outcomes and overall quality of life for patients battling hematological cancers.

### 4.6. Strengths and Limitations of the Present Study

A notable strength is the comprehensive data collection process, which involved an extensive literature search across major databases like MEDLINE and SCOPUS. This thorough search ensured a wide-ranging collection of relevant studies, culminating in the inclusion of 25 articles that offer a detailed overview of the topic. The diversity in study designs, encompassing both cross-sectional and longitudinal studies, provided insights into cognitive changes at both single time points and over time. Additionally, the global representation of the research, including contributions from the United States, Europe, Brazil, Asia, Israel, Egypt, and Australia, allowed for a nuanced understanding of cognitive impacts across different patient demographics and healthcare settings. The study’s detailed analysis of neuropsychological testing tools further highlighted specific cognitive domains affected by these malignancies, offering valuable information on the methodologies used.

However, the study also faced several limitations. The heterogeneity among the included studies, in terms of patient characteristics, types of hematologic malignancies, and methodological approaches, posed challenges. This variability prevented the authors from performing a statistical or meta-analysis, potentially affecting the generalizability of the findings. One of the key limitations of this review is the lack of control groups in 60% of the included studies, which weakens the ability to draw definitive comparisons between the cognitive impacts of hematologic malignancies and the general population. Studies with control groups [17,31] have provided valuable insights, demonstrating worse cognitive performance in patients with myelodysplastic syndromes compared to healthy controls. However, the absence of control groups in many studies highlights the need for future research to include well-matched controls to enable more robust comparisons. Moreover, the review’s focus on English-language articles introduced a potential language bias, as it excluded studies published in other languages and did not consider unpublished studies or grey literature, possibly omitting relevant data. The variability in the timing of neuropsychological assessments across studies, ranging from baseline to post-treatment and long-term follow-ups, could lead to inconsistencies in detecting cognitive changes over time. Lastly, the study identified a need for more longitudinal data to understand the long-term cognitive effects of treatments, highlighting a gap in the current literature.

### 4.7. Future Implications

Future research should focus on refining cognitive assessment tools that are sensitive to the specific needs of patients with hematologic malignancies. The development of standardized protocols that include biomarkers, such as inflammatory markers and hemoglobin levels, could provide deeper insights into the cognitive status and help predict potential declines. Moreover, there is a need for longitudinal studies to track cognitive changes over extended periods, which would provide valuable data on long-term cognitive outcomes and the effectiveness of various interventions. The healthcare community must prioritize training for healthcare providers to recognize and manage cognitive impairments. This includes developing and implementing targeted cognitive rehabilitation programs and holistic care strategies that consider psychological and social aspects. Future studies should also explore protective strategies against cognitive decline, particularly in the context of chemotherapy and other intensive treatments. Moreover, addressing the under-recognition and undertreatment of cognitive deficits is crucial. Efforts should be made to ensure that cognitive impairments are not overshadowed by other physical health concerns, particularly in older adults, where symptoms may be mistakenly attributed to aging. By prioritizing cognitive health, healthcare providers can significantly improve the overall quality of life for patients with hematologic malignancies. Finally, future systematic reviews and meta-analyses may also examine the cognitive profiles in MDS as well as other specific hematological malignancies by systematically addressing the role of demographic factors and other clinical-related variables (such as inflammation, specific treatments) and examining long-term outcomes, further attempting to reach conclusions which may assist in the formation of guidelines for the best course of management for patients with MDS and other hematological malignancies.

## 5. Conclusions

In conclusion, this scoping review provides a comprehensive examination of the cognitive impairments associated with hematologic malignancies, particularly MDS and AML. The findings underscore the significant prevalence and diversity of cognitive deficits in these patient populations, highlighting the impact of factors such as age, disease type, and treatment modalities. The review also reveals the complex interplay between systemic inflammation and cognitive decline, suggesting potential therapeutic targets. Despite the valuable insights gained, the study is limited by heterogeneity among included studies and a lack of standardized assessment protocols. Future research should aim to develop refined cognitive assessment tools and establish standardized protocols, including biomarkers, to better understand and manage cognitive impairments in these patients. Such advancements are crucial for improving patient care and quality of life, ensuring that cognitive health is prioritized alongside physical health in the management of hematologic malignancies.

## Figures and Tables

**Table 1:** Quantitative Summary for the Results Section.

Parameter	Descriptive Data
Total Studies	25
Study Type	Longitudinal: 17 (68%), Cross-sectional: 8 (32%)
Mean Age (SD)	63.9 years (12.8)
Control Groups Included	40% (10 studies)
Cognitive Domains Assessed	Executive Function (68%), Memory (56%), Attention (44%)
Impairment Rates Observed	17% to 70.6%
Treatment-Specific Findings	HSCT: 6 studies, Chemotherapy: 4 studies

## Data Availability

No new data were created.

## Supplementary Materials

The following supporting information can be downloaded at: https://www.mdpi.com/article/10.3390/medsci13010015/s1, Table S1: Basic characteristics and main findings of the included studies (presented in chronological order, from the oldest to the newest) https://doi.org/10.5281/zenodo.14745231 (accessed on 20 December 2024).
