# Supplementary material for: A Scoping Review on Cognition in Myelodysplastic Syndromes: Advances and Challenges
Source: Med Sci (Basel). 2025 Feb 7;13(1):15. doi: 10.3390/medsci13010015 (PMC11843857; doi:10.3390/medsci13010015)
Supplement: Supplementary file 1 [file medsci-13-00015-s001.zip › medsci-3416206-supplementary.pdf]

Supplementary Table S1. Basic characteristics and main findings of the included studies (presented in chronological order, from the oldest to the newest)
https://doi.org/10.5281/zenodo.14745231

First author (Year)	Study Type / Study Origin	Patients (N, Gender, Age)	Controls (N, Gender, Age)	Time of NPS exam / Neuropsychological tests	Main findings on cognition
Beloosesky (2000)	L / Israel	• 37<br>• 22 F / 15 M<br>• 81.3 years	N/A	• Range of follow-up period between 1–70 months<br>• MMSE; iADL	• No differences between demented and cognitively normal patients in age, sex, comorbidities or laboratory parameters.<br>• Only dementia adversely affects survival, compared to cognitively normal status.<br>• In contrast to the hematological parameters, the cognitive level had a clearly more important impact.<br>• As in the general population, cognitively normal patients live significantly longer than demented patients
Meyers (2005)	L / USA	• 54 (35 MDS)<br>• 24 F / 30 M<br>• 60.2 years	N/A	• Pre-treatment evaluation and 1 month later<br>• WAIS Digit Span; WAIS Digit Symbol; HVLT; COWAT; TMT Part A&B; Grooved Pegboard	• A significant proportion of patients had impaired cognitive function prior to chemotherapy.<br>• Significant fatigue in 65%<br>• Higher IL-6 levels were associated with poorer executive function<br>• Higher IL-8 levels were associated with better memory performance.<br>• IL-6, IL-1RA, and TNF-levels were related to ratings of fatigue.<br>• Fatigue and cognitive dysfunction were unrelated.<br>• Hemoglobin levels were not significantly associated with cognition or fatigue.<br>• Treatment did not significantly impact cognition.
Poppelreuter (2008)	L / Europe	• 75 (3 MDS)<br>• 37 F / 38 M<br>• 42.59 years	N	• At the start of in-patient rehabilitation (T1), at the end of	• A total of 79.5% patients presented signs of clinically relevant deficits in at least one of 16

				rehabilitation (T2), and 6 months later (T3)<br>• TAP; EORTC QLQ-C30; MFI; FEDA	neuropsychological parameters, with 50.7% showing deficits in two or more.<br>• Allogeneic HSCT and female gender were risk factors for neuropsychological impairment.<br>• Significant improvement in neuropsychological parameters between T1 and T2 in both intervention groups and in the control group as well<br>• A total of 35.5% of patients demonstrated deficient performance in at least one neuropsychological parameter at T3, with 22.6% showing deficits in two or more parameters.<br>• Alertness, divided attention, sustained attention and verbal-semantic memory were the parameters showing the highest rates of deficits at all the measured time points.
Schulz-Kindermann (2007)	L / Europe	• 39 (4 MDS)<br>• 14 F / 25 M<br>• 45.6 years	N/A	• 2 weeks before admission (baseline T0), and 100 days (range: 80–120 days) after transplantation (T1)<br>• TMT Parts A&B; TAP; WMS Digit Span and Visual Memory Span; AVLT; Regensburg Word Fluency Test; Achievement Measure System	• Verbal long-term memory was below the norm population, whereas reasoning was significantly better than the norm.<br>• At 100 days post HSCT, another test parameter of verbal long-term memory was below norm.<br>• Visual working memory measured showed a mean below norm; reasoning exceeded population norms significantly.<br>• A significant decline in cognitive performance was observed in simple reaction time.<br>• Regarding executive functions, no patient displayed impairment of reasoning at either point in time.<br>• 21.1% of patients demonstrated no cognitive impairment at T0 and T1.<br>• Ten patients (52.6%) showed impairments in 1–3 functions (‘mild impairment’) at both assessments.

					• According to a definition of 'global impairment' (i.e. four and more abnormal functions), five patients (26.3%) were rated as globally cognitively impaired at T0 and the same percentage was found at T1.<br>• Regarding intraclass changes, 17 of 19 patients showed stable performance and were ranging in the same class of impairment (no, mild, global) at both time points, one patient improved from 'global' to 'mild' impairment, and another one declined from 'mild' to 'global' impairment.
Chang (2009)	L / USA	• 15<br>• 4 F / 11 M<br>• 64.8 years	N/A	• At enrollment and then, 12 months and 18 months after HSCT<br>• TMT Part A&B; VFT; BSRT; Grooved Pegboard	• Among all patients, there was significant improvement in memory over 18 months.<br>• Patients with CML overall had improved phonemic fluency.
Wood (2011)	C / USA	• 88 (47 MDS)<br>• 37 F / 51 M<br>• 61 years	N/A	• hemoglobin levels were measured within one day of the neuropsychological assessments<br>• WAIS Digit Span and Digit Symbol; HVLT; TMT Part A &B; COWAT	• Cognitive impairment in 47% of patients with MDS compared to 30% with other hematologic malignancies.<br>• Specific deficits included executive dysfunction and memory impairments, with the most significant effects observed in patients undergoing intensive treatments.
Scherwath (2012)	L / Europe	• 239 (33 MDS)<br>• 91 F / 148 M<br>• 50.4 years	N/A	• Before conditioning/at admission (T0), 100 days after (T1), and 12 months after allogenic HSCT (T2)<br>• TAP; WMS Digit span and Visual span; VLMT; Regensburg Word Fluency Test; Grooved Pegboard Test; the Multiple Choice Knowledge Test-B	• Before and after HSCT, patients performed below test norms in up to 50% of the test scores.<br>• Patients were mostly impaired on word fluency (24%, T0), fine motor function, and VLMT delayed recall (19% each, T2).<br>• Impairment on ≥1/5 cognitive domains occurred in 47% (T0) and 41% (T2) of the patients.<br>• Performance partially improved over time (i.e., WMS visual span forward, VLMT, and word fluency).

					• From baseline to T2, 16% of the patients showed reliable decline on ≥3/14 test scores.<br>• For most neuropsychological subtests, no associations with conditioning intensity, total body irradiation, graft-versus-host disease, cyclosporine treatment, and length of hospital stay were found.<br>• Age and premorbid intelligence level were consistently associated with cognition.
Hamaker (2013)	L / Europe	• 108 (25 MDS)<br>• 51 F / 57 M<br>• 78.2 years	N/A	• In hospital (newly diagnosed)<br>• G8 questionnaire; MMSE; GDS; Barthel index; IADL; F-Sozu	• High prevalence of geriatric conditions with:<br>•cognitive dysfunction in 17%<br>• symptoms of depression in 24%,
Castelli (2014)	L / Europe	• 24<br>• 10 F / 14 M<br>• 72 years	N/A	• In basal condition and 8 weeks after treatment<br>• MMSE; FACT-An	• A positive and significant correlation between MMSE and Hb scores
Chang (2014)	L / USA	• 106 (15 MDS)<br>• 48 F / 58 M<br>• 47.9 years	N/A	• At the initiation of the treatment and then, after 12 months<br>• BSRT; WAIS Digit Span and Digit Symbol; TMT Part A and B; VFT; SCWT; Grooved Pegboard.	• Semantic fluency was the only cognitive measure associated with change in the QoL aspect measured by the mental component summary scale of the Medical Outcomes Study SF−36.<br>•
Hoogland (2017)	L / USA	• 140 (27 MDS)<br>• 59 F / 81 M<br>• 51.12 years	• 75<br>• 42 F / 33 M<br>• 52.97 years	• Before transplantation and 3 months and 1 year after transplantation<br>• WTAR; BVMT; HVLT; COWAT; WAIS Digit Span; Color Trails Test Part 1&2; SNST	• HCT recipients demonstrated worse performance than non-cancer controls before transplantation in verbal memory, visual memory, and total neuropsychological performance.<br>• HCT recipients demonstrated worse performance than controls over time in executive functioning.<br>• Patients age ≥65 years demonstrated worse verbal memory and verbal fluency than older and younger (<65 years) controls post-transplantation.
Hoogland (2019)	L / USA	• 85 (15 MDS)<br>• 36 F / 49 M	N/A	• Pre-HCT and 90 days after HCT	• Increased IL-6 and sTNF-RII from pre-HCT to 90 days after HCT were associated

		• 52.0 years		• WTAR; HVLT; COWAT; BVMT; WAIS Digit Span; Color Trails Test; SNST; ECog scale	with declines in TNP, verbal memory, attention, and executive functioning.<br>• Decreased CRP over time was associated with better TNP, verbal memory, and visual memory.<br>• No associations between systemic inflammation and subjective cognition.
Koll (2019)	L / USA	• 51 (13 MDS)<br>• 17 F / 34 M<br>• 68.31 years	N/A	• Before and after HCT<br>• MoCA; Phonemic Verbal Fluency; BNT; WLL; Praxis 2D and 3D; TMT Part A and B	• The prevalence of cognitive impairment detected by neuropsychological tests ranged between 53% to 70.6% using the criteria for patients with cancer by ICCTF.<br>• The following cognitive domains were most affected: language, learning and memory, visuospatial skills, and executive function.<br>• MoCA is an appropriate screening test for cognitive impairment.<br>• Using the ICCTF criteria, 86 to 100% of patients are correctly classified as having significant cognitive impairment on neuropsychological tests using a cut-off score of 20 or less.
Kotb (2019)	C / Egypt	• 150<br>• 53 F / 97 M<br>• 45.53 years	N/A	• 6 months to 2 years after the end of chemotherapy<br>• MoCA	• Out of 150 patients with different hematological malignancies who finished their chemotherapy at least 6 months ago, 93 patients (62%) were cognitively impaired.<br>• The average score of MoCA for all patients was 23.913 ± 3.997.<br>• CICI is more among patients who received parenteral chemotherapies and closely related to premedication comorbidities, and all patients (MDS) were cognitively impaired.<br>• There was a positive correlation between patient age and cognitive handicap as mean age of patients with abnormal cognitive function, while period of hospital admission was showing significant

					correlation with impaired abstraction function, and number of chemotherapy cycles showed significant correlation with naming and orientation cognitive impairment<br>• Female patients had significant shortcoming in naming ingredient more than male.<br>• The type of chemotherapy regimen received did not significantly affect the overall cognitive impairment, but patients who had received Velcade-based chemotherapy had significantly lower executive and abstract function<br>• Patients which did not achieve remission at follow-up have markedly significant lower scores of most of the cognitive social function.
LaLonde (2019)	L / USA	• 46 (6 MDS)<br>• 22 F / 24 M<br>• 55.78 years	N/A	• Pre-HSCT and at 30 and 100 days post-HSCT<br>• CogState; FACT-Cog; PHQ-9; GAD-7; PSQI; FACT-G	• Patient-reported cognitive complaints were largely independent of concurrently assessed objective neuropsychological performance.<br>• Significant medium to large effect size associations between subjective cognitive complaints post-HSCT with objectively measured change from pre-HSCT in attention, visual learning, and working memory.<br>• Subjective cognitive complaints post-HSCT were associated with depression, anxiety, daytime sleepiness and physical well-being.
Molga (2019)	C / Australia	• 98 (91 MDS)<br>• 36 F / 62 M<br>• 77 years	N/A	• After the treatment decision was made<br>• IADL; TGUG; "Falls" questionnaire; MMSE; GDS; MNA; CCI ; CGA	• Patients had deficits in at least one CGA domain.<br>• Deficits were spread across all CGA domains, including dependence for instrumental activities of daily living.<br>• Patients who were dependent for IADL, had cognitive impairment or impaired mobility completed significantly fewer azacitidine cycles as compared to those without these deficits.

					• Cox-proportional regression showed that IADL dependency and higher comorbidities were associated with poor prognosis independent of disease related factors.
Rodrigues (2019)	C / Brazil	• 40 (16 MDS)<br>• 26 F / 14 M<br>• 67.6 years	N/A	• In hospital (at least one week before admission for allo-HSCT)<br>• IADL; MMSE; VFT; Clock Test; GDS	• Normal MMSE in 89%.<br>• The clock test and VFT were performed in only 27 of the 40 patients.<br>•Clock test was normal only in 15%<br>•VFT was normal in 70.4%.<br>•Complaints for loss of memory in 37.5%.<br>•GDS scale was normal in 82.5%.<br>• Normal GDS scores in 82.5%
Kim (2020)	L / Asia	• 41 (9 MDS)<br>• 18 F / 23 M<br>• 48.63 years	N/A	• At Enrollment and at discharge<br>• DEMMI; VAS; EORTC QLQ-C30; Zung-SDS.	• No significant differences in EORTC QLQ-C30, including physical functioning, emotional functioning, and cognitive functioning at admission, and physical, role, emotional, and cognitive functioning at discharge between the groups, in which the patients with physical impairment had significantly lower scores.<br>• A significant difference in Zung-SDS between the groups at discharge.
Nagl (2020)	C / Europe	• 209 (48 MDS)<br>• 98 F / 111 M<br>• 77.2 years	N/A	• Within 14 days after initial diagnosis<br>• MMSE; WHO-PS; Karnofsky Performance Score, Barthel Index, IADL, GDS	• Comorbidities have a great prognostic impact, independently deteriorating survival and clustering with impairments, namely reduced functional and objective physical capacities, impaired performance, and depressive mood.
Nakamura (2020)	C / USA	• 65 (12 MDS)<br>• 26 F / 29 M<br>• 54.2 years	N/A	• After admission to the unit and prior to transplantation<br>• MoCA	• Cognitive impairment (MoCA < 26) in over 50% of patients prior to transplantation.<br>• When adjusted for demographic variables, two characteristics were significantly associated with worse cognitive function: the hematopoietic cell transplantation-comorbidity index score and history of alcohol or substance abuse.

					• Pre-HSCT cancer and cancer treatment–specific variables were not associated with cognitive function.
Wall (2021)	C / USA	• 311 (47 MDS)<br>• 138 F / 173 M<br>• 76 years	N/A	• When referred to the clinic<br>• BOMC test or MoCA	• One-third of patients (n = 95, 33%) had cognitive impairment based on BOMC or MOCA.
Lew (2022)	L / USA	• 157 (42 MDS)<br>• 56 F / 98 M<br>• 18–60 years	N/A	• At new patient evaluation, final visit at approximately 1 week prior to HCT<br>• MoCA; PHQ9; PC-PTSD-5	• Cognitive impairments occurred in 58 patients (36.9%)<br>• Patients aged 18 to 39 years were more likely to have a higher average MoCA score than patients aged 40 to 59 years and patients age ≥60 years.<br>• Patients who did not make it to HCT were more likely to have cognitive impairment (22 of 44 (50.0%) versus 24 of 77 (31.2%)) and psychological impairment (16 of 44 (36.4%) versus 15 of 77 (19.5%));<br>• Patients aged ≥60 years were more likely to have a cognitive impairment at SO (12 of 31; 38.7%) compared with patients aged 18 to 39 years (1 of 20; 5.0%) and those aged 40 to 59 years (5 of 26; 19.2%)<br>• From NPE-SO: of the 18 with impairments in cognitive function, 9 (50.0%) improved
Loh (2023)	L / USA	• 20 (8 MDS)<br>• 7 F / 13 M<br>• 71.2 years	N/A	• Pre- and post-intervention<br>• MoCA; Grip strength	• At baseline DunedinPACE was inversely correlated with grip strength.
Meadows (2023)	L / USA	• 77 (15 MDS)<br>• 37 F / 40 M<br>• 48.6 years	N/A	• At baseline, 12 months, and 18 months post-treatment<br>• WAIS Digit Span and Digit Symbol; TMT Part A &B; VFT; SCWT; BSRT; Grooved Pegboard	• At baseline, 23% of the participants in the longitudinal sample had Z-scores on at least one of the neuropsychological tests that Z ≤ -1.4.<br>• Participants showed improvement from baseline to follow-up assessments.

					• The average Z-scores for the six cognitive domains in the longitudinal data set over the course of the study ranged from 20.89 to 0.59.<br>• Significant predictors of neuropsychological change included age, with older participants showing less improvement over time.<br>• Other predictors included baseline cognitive scores (language, memory, and attention), previous cocaine use, disease status, intelligence quotient, and quality of life measures.
Wauben (2023)	C / Europe	• 115 (6 MDS)<br>• 50 F / 65 M<br>• 58.4 years	• 465<br>• 257 M/208 F<br>• 58.9 years	• Patients who survived at least 2 years after autologous or allogeneic stem cell transplantation without evidence of recurrent or active disease<br>• VLT; LDST; CST Parts A&B&C; SCWT Parts I–III	• The prevalence of cognitive dysfunction was 34.8% in HCT survivors and 21.3% in the reference group.<br>• When adjusted for age, sex, and level of education, HCT survivors had a worse overall cognition score, translating into 9.0 years of higher cognitive age.<br>• HCT survivors scored worse on memory, information processing speed and executive function and attention than the reference group.<br>• The odds of cognitive impairment were on average 2.4 times higher among HCT survivors than the reference group.<br>• Within the HCT survivor group none of the tested clinical determinants of cognitive impairment were significantly associated with cognition.

Notes. L: longitudinal; C: cross-sectional; F: Female; M: Male; MDS: Number of patients with Myelodysplastic syndrome; N: total number; N/A: not applicable; IADL: Instrumental Activities of Daily Living; NPS: Neuropsychological; HSCT: Hematopoietic Stem Cell Transplantation; HCT: Hematopoietic Cell Transplantation; TNP: total neuropsychological performance; CRP: C-reactive protein; IL-6=interleukin-6; sTNF-RII=soluble tumor necrosis factor receptor 2; IL-1RA: receptor antagonist; TNF: tumor necrosis factor; AML: Acute Myeloid Leukemia; NPE: new patient evaluation; SO: sign-off; Hb: hemoglobin; ER: erythroid response; CICI: Chemotherapy-induced cognitive impairment; CML: chronic myelogenous leukemia; CGA: Comprehensive Geriatric Assessment; MMSE: Mini-Mental State Examination; GDS: Geriatric depression scale; F-Sozu: Fragebogen zur Erfassung der sozialen Unterstützung; IADL: Instrumental activities of daily living; VFT: Verbal Fluency Test; DEMMI: The De Morton Mobility Index—

physical functioning; VAS: The Visual Analogue Scale; EORTC QLQ-C30: European Organization for Research and Treatment of Cancer quality of life questionnaire; MFI: Multidimensional Fatigue Inventory; FEDA: Fragebogen erlebter Defizite der Aufmerksamkeit; Zung-SDS: Zung Self-rating Depression Scale; WTAR: Wechsler Test of Adult Reading; HVLT: Hopkins Verbal Learning Test—Revised Immediate and Delayed Recall; COWAT: Controlled Oral Word Association Test ; BVMT: Brief Visuospatial Memory Test—Revised Immediate and Delayed Recall; WAIS: Wechsler Adult Intelligence Scale; SNST: Stroop Neuropsychological Screening Test; ECog: Everyday Cognition scale; TMT: Trail Making Test Part A&B; TAP: Tests of Attentional Performance (TAP); WMS-R: Wechsler Memory Scale—Revised; VLMT: Verbal Learning and Memory Test (VLMT); MoCA: Montreal Cognitive Assessment; PHQ-9: Patient Health Questionnaire 9;

PC-PTSD-5: The Primary Care PTSD Screen for DSM 5; FACT-An: The Functional Assessment of Cancer Treatment—Anemia; WHO-PS: World Health Organization Performance Score; iADL: The index of activities of daily living; BNT: Boston Naming Test; WLL: Word List Learning; BSRT: Buschke Selective Reminding Test; SCWT: Stroop Color Word Test; BOMC: The Blessed Orientation Memory and Concentration test; FACT-Cog: Functional Assessment of Cancer Therapy—Cognitive Scale version 3; GAD-7: General Anxiety Disorder-7; PSQI: Pittsburgh Sleep Quality Index; FACT-G: Functional Assessment of Cancer Therapy—General; VLT: The Verbal Learning Test; LDST: the Letter-Digit Substitution test; CST: Concept Shifting Tests; AVLT: Auditory-Verbal-Learning-Test; TGUG: Timed Get Up and Go; MNA: Mini Nutritional Assessment; CCI: Charlson Comorbidity Index; ICCTF: International Cancer Cognition Task Force.
